# Detection and identification of enteroviruses circulating in children with acute gastroenteritis in Pará State, Northern Brazil (2010–2011)

**DOI:** 10.1186/s12985-020-01431-w

**Published:** 2020-10-16

**Authors:** Raiana Scerni Machado, Ivanildo Pedro de Sousa, Jacqueline Cortinhas Monteiro, James Lima Ferreira, Jainara Cristina dos Santos Alves, Fernando Neto Tavares

**Affiliations:** 1 Laboratório de Referência Regional em Enteroviroses, Seção de Virologia, Instituto Evandro Chagas, Ananindeua, Pará Brasil; 2 Laboratório de Enterovírus, Instituto Oswaldo Cruz, Fundação Oswaldo Cruz, Rio de Janeiro, Brasil; 3 Laboratório de Virologia, Instituto de Ciência Biológicas, Universidade Federal do Pará, Belém, Pará Brasil

**Keywords:** Enterovirus, Acute gastroenteritis, Brazil

## Abstract

Although acute gastroenteritis (AGE) has been reported as a common infectious disease in children, there is scarce information about enterovirus (EV) circulating associated with AGE cases in Brazil. The purpose of the present study was to identify and characterize the enteroviruses associated with AGE in children in Belém, Brazil. A total of 175 stool samples were obtained from children hospitalized revealing the presence of EV in 26.3% (46/175) of infections. EV type was identified in 78.3% (36/46) and EV-B species (61.1%; 22/36) was the most prevalent EV-detected followed by EV-C (25%; 9/36) and EV-A (13.9%; 5/36). This study has provided important information about the enterovirus circulation in Pará state, Northern Brazil.

Acute gastroenteritis (AGE) is one of the most common diseases in humans, mainly in children and remains as important cause of morbidity and mortality among infants around the world [1]. Children under 5 years old are the most affected with highest incidence and leading cause of million deaths annually worldwide, occurring mainly in low as well as in middle-income countries [2]. In Brazil, AGE also presents higher morbidity rates representing a significant cause of death in the first year of life [3–5]. AGE can be caused by a variety of infectious agents (viral, bacterial, protozoan) as well as non-infectious agents [4]. Among the viral agent, rotavirus, calicivirus, norovirus, adenovirus and astrovirus have been demonstrated as the most frequent causes of AGE in children [6, 7]. Recently, different members of the *Picornaviridae* family, such as *Parechovirus, Cosavirus, Salivirus and Aichivirus* have been identified as agents associated with diarrhea in humans [8, 9]. The *Picornaviridae* family also has the *Enterovirus* genus, whose association with AGE has been recognized and reported in many studies [1, 7, 10]. Although enteroviruses (EV) infections are mostly asymptomatic, these viral agents can cause severe infection, such as syndromes of the central nervous system, myocarditis and neonatal sepsis [10].

In Brazil, a limited number of studies on EV that are associated with AGE have been reported [11, 12]. These works were based on description only one specific type, hence, the epidemiological analysis of EV infection in AGE patients is restricted. Thus, the purpose of this study was to identify the circulating genotypes of EV isolated from children with AGE symptoms in Belém (Pará state), Northern region from Brazil providing valuable information about EV circulation.

From May 2010 through April 2011, 175 stool specimens were collected from children (< 5 years) who had been suffering from AGE and attended to the Pediatric Clinic of Pará. Viral RNA was extracted (Viral Nucleic Acid Extraction Kit-QIAmp-Qiagen) directly from the clinical specimens and initially subjected to a broad-reactive real time RT-PCR (rRT-PCR) for human enteroviruses as previously described [13, 14]. EV-positive samples (46/175; 26.3%) in the rRT-PCR were submitted to viral isolation in RD and HEp2-C cell lines and incubated at 37ºC and examined daily for cytopathic effect (CPE) with total destruction of the cell monolayer, which is a characteristics of enterovirus infection. Conventional PCR was performed using a pair of primers (222 and 292) that amplifies a fragment of approximately 350 bp within the VP1 gene, as described [14, 15].

After inoculation, the specimens that did not have effect in the production of CPE (30/46; 65.2%) were submitted to a semi-nested PCR (RT-snPCR) amplification of partial VP1 gene according to previously described [14, 16]. EV-positive amplicons from RT-sn PCR (22/30; 73.3%) and conventional PCR/cell culture (16/46; 34.8%) (Table 1) were cycle-sequenced by the Sanger method using a BigDye Terminator v3.1 Cycle Sequencing Kit (Applied Biosystems), and the nucleotide sequences obtained were compared with those available in the GenBank database to determine the viral types. In general, we were able to identify EV type in 78.3% (36/46) of the samples, which revealed a detection pattern EV-B (61.1%; 22/36) > EV-C (25%; 9/36) > EV-A (13.9%; 5/36) (Table 2). These findings were similar to previous reports that showed EV-B species more frequently detected than EV-C and EV-A species in children with AGE [18–20]. Some EV positive samples in rRT-PCR could not be typed (8/46; 17.4%) due to failure to produce amplicons. Additionally, two other specimens showed a problem in genotyping. Sequences of the primers used in this study for EV detection are shown in Table 3. Noteworthy that the specimens were tested for other viral agents, such as rotavirus, parechovirus and aichivirus and the co-infection EV and non-enterovirus was observed with aichivirus.

**Table 1:** Specimens used in this study obtained from patients with AGE symptoms, and laboratory results

Type of specimens	N. specimens	Results by: rRT-PCR;cell culture; RTsn-PCR (positive/specimens)	EV positive	EV positive rates (%)
Stool	175	46/175; 16/46; 22/30	46	26.3 (46/175)

**Table 2:** Enterovirus types associated with AGE in children hospitalized in Pará State, Brazil

Species	Type	N. total (% of total)
A	CVA5	2 (5.5)
	CVA6	2 (5.5)
	CVA10	1 (2.7)
Subtotal		5 (13.9)
B	CVA9	1 (2.7)
	CVB3	4 (11.1)
	CVB4	2 (5.5)
	E3	1 (2.7)
	E6	1 (2.7)
	E7	4 (11.1)
	E9	4 (11.1)
	E14	1 (2.7)
	E15	2 (5.5)
	E18	1 (2.7)
	E25	1 (2.7)
Subtotal		22 (61.1)
C	CVA13	2 (5.5)
	EV-C96	1 (2.7)
	EV-C99	3 (8.3)
	PV1	1 (2.7)
	PV3	2 (5.5)
Subtotal		9 (25)
Total		36 (100)

**Table 3:** Sequences of the Primers used in Real-Time PCR, cDNA synthesis, PCR amplification and sequencing for EV detection

Primer/ Probe	Sequence^a^	Gene	Location^b^
EVReal T(A) ^c^	GCGATTGTCACCATWAGCAGYCA	5′-UTR	599–577
EVRealT(S)^**c**^	GGCCCCTGAATGCGGCTAATCC	5′-UTR	449–470
PanEVProbe (S)^**c**^	FAM-CCGACTACTTTGGGWGTCCGTGT-MGBNFQ	5′-UTR	537–559
AN32^d^	GTYTGCCA	VP1	3009–3002
AN33 ^d^	GAYTGCCA	VP1	3009–3002
AN34 ^d^	CCRTCRTA	VP1	3111–3104
AN35 ^d^	RCTYTGCCA	VP1	3009–3002
222 ^d^	CICCIGGIGGIAYRWACAT	VP1	1977–1996
224 ^d^	GCIATGYTIGGIACICAYRT	VP3	2969–2951
292 ^d^	MIGCIGYIGARACNGG	VP1	2612–2627
AN88 ^d^	TACTGGACCACCTGGNGGNAYRWACAT	VP1	2977–2951
AN89 ^d^	CCAGCACTGACAGCAGYNGARAYNGG	VP1	2602–2627

^a^Degenerate primers: Y = C or T; R = A or G; W = A or T; M = A or C; N = A, C, G or T; I = Inosine;

^b^The locations of all primers are relative to the genome of poliovirus type 1, Mahoney strain;

^c^[17];

^d^[14, 16]

CVB3, E7 and E9 were the most frequently detected type from the EV-positive specimens (Table 2). Furthermore, it is worth mentioning the high detection rate of EV-C species and the identification of uncommon types, such as EV-C99 and EV-C96, mainly identified in Hep2C cell line. These results suggest the importance of the Hep2C cell culture in the NPEVs surveillance as previously reported, which can favor an increased number of EV-C isolates [21].

In this study, 26.3% (46/175) of specimens analyzed were EV-positive. Similar results were obtained from studies performed in India, which reported an EV detection rate of 33–40% [22]. Surprisingly, although it has been shown that enteroviruses can be associated with AGE cases in children, the high detection rate as observed in this study is not common. This difference can be due to viral detection methods and seasonal factors. This result suggests that the circulating of these viruses may not be well known in children in Belém.

To further analyze EV types identified in AGE patients, we carried out a phylogenetic analysis based on partial VP1 sequences (Fig. 1). CVA5, CVA6 and CVA10 strains circulating in Belém were closely related to viruses detected in Japan, China and The Netherlands (Fig. 1a). Regarding EV-B species, the strains evaluated in this study were classified as shown in Fig. 1b. The Brazilian sequences were grouped into different clusters according to type and had a relative close relationship with previously strains circulating mainly in Europe and Asian (Fig. 1b). The phylogenetic analysis of the EV-C species isolates of this study revealed that the Brazilian strains were related to viruses previously circulating in China, Finland and Uruguay (Fig. 1c). The polioviruses found in this work (PV1 and PV3) were analyzed to a nucleotide divergence and revealed a high homology to Sabin-like viruses (Fig. 1c).

**Fig. 1:**
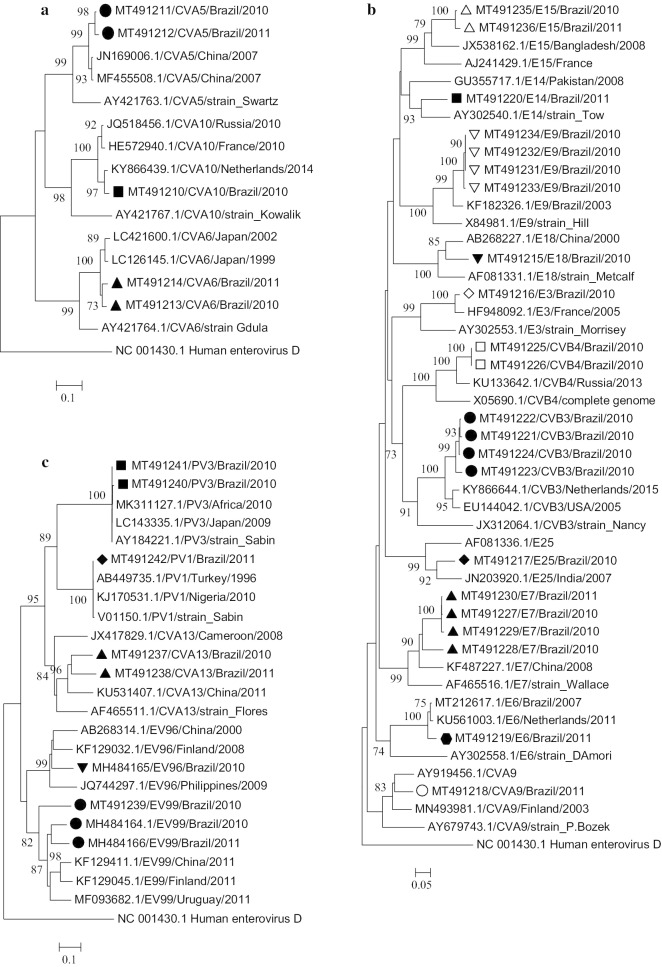
Phylogenetic analysis based on partial VP1 sequences (~ 300 bp) from Brazilian isolates associated with AGE cases and other sequences available at the GenBank database. Phylogenetic trees were constructed with MEGA 6.0 software by the Neighbor Joining method using Kimura 2-parameter substitution model and validated with 1000 pseudo-replicates. Only bootstrap values > 70% are shown at the node. Geometric shapes indicate the enteroviruses identified in this study. **a** EV-A species, **b** EV-B species and **c** EV-C species

## Conclusion

Overall, this work provides valuable information about the circulation and the genetic diversity of EV associated with AGE cases, reinforcing the need of tailoring current surveillance strategies to timely monitor emergence/re-emergence of non-polio enteroviruses. Furthermore, the data obtained from monitoring of diarrhea cases can reveal important information on the PV circulation (Sabin, VDPV or wild type) in areas of low vaccine coverage and deficient acute flaccid paralysis surveillance. Additionally, in the context of global eradication of polioviruses, information on non-polio enteroviruses circulation is key to understand their role in AGE context and other enteroviruses infections-associated. The difficult in the access the patient records and to survey other causative agents involved in AGE represented a study limitation.

## Data Availability

The data analyzed during the current study are available from the corresponding author on reasonable request.

## Abbreviations

AGE: Acute gastroenteritis
EV: Enterovirus
CV: Coxsackievirus
E: Echovirus
PV: Poliovirus
VDPV: Vaccine-derived poliovirus
